# Rule-Based Ion Prediction with Orthogonal Constraints Reveals Bacterial Phospholipid Remodeling Signatures

**DOI:** 10.3390/antibiotics15050459

**Published:** 2026-04-30

**Authors:** Wanying Hu, Wenhan Li, Meirong Song, Jianfei Zhu, Kui Zhu

**Affiliations:** 1 State Key Laboratory of Veterinary Public Health and Safety, College of Veterinary Medicine, China Agricultural University, Beijing 100193, China; b20223050397@cau.edu.cn (W.H.); liwh@cau.edu.cn (W.L.); meirong_song@cau.edu.cn (M.S.); zhujf@cau.edu.cn (J.Z.); 2 Engineering Research Center of Animal Innovative Drugs and Safety Evaluation, Ministry of Education, College of Veterinary Medicine, China Agricultural University, Beijing 100193, China; 3 Technology Innovation Center for Food Safety Surveillance and Detection (Hainan), Sanya Institute of China Agricultural University, Sanya 572025, China

**Keywords:** bacterial phospholipid profiling, membrane remodeling, antibiotic tolerance, MS/MS, Paternò–Büchi derivatization

## Abstract

**Background:** Phospholipids are essential components of bacterial membranes and play central roles in membrane integrity and adaptation to antibiotic stress. However, confident annotation of phospholipid molecular species remains challenging due to the complexity of the lipidome and the limited structural constraints in conventional lipidomics workflows. **Methods:** Here, we present a bacterial phospholipidomic framework that integrates orthogonal structural evidence to achieve high-confidence and traceable annotation. Thin-layer chromatography (TLC) provides phospholipid headgroup assignment, gas chromatography–mass spectrometry (GC–MS) defines the acyl-chain pool, and Paternò–Büchi derivatization enables C=C localization, collectively restricting the structural search space prior to liquid chromatography–tandem mass spectrometry (LC–MS/MS) analysis. A rule-based ion prediction library further standardizes diagnostic ion assignment and reduces annotation ambiguity. **Results:** Applying this platform, we found *Escherichia coli* in the stationary phase remodeled the membrane phospholipids, with cardiolipin (CL) increasing from ~5% to ~10% and cyclopropane-containing phospholipid species rising to ~75%. Similar remodeling patterns are observed under diverse antibiotic exposures at sub-inhibitory concentrations, consistent with convergence toward a tolerance-associated membrane state. Extension of the framework to *Enterococcus faecium* supports proof-of-concept application in an additional Gram-positive model, with vancomycin-resistant strains exhibiting pronounced phosphatidylglycerol (PG) enrichment and reduced CL. **Conclusions:** Our work provides a scalable and reproducible strategy for bacterial phospholipid annotation, enabling molecular-species-resolved investigation of membrane adaptation and offering a framework for future exploration of lipid homeostasis pathways as potential antimicrobial targets.

## 1. Introduction

Phospholipids form the structural basis of bacterial membranes and are continuously remodeled to tune permeability, lateral packing, curvature stress, and the membrane environment to ensure essential protein machinery [1,2,3,4,5,6]. This remodeling is encoded not only in headgroup composition but also in acyl-chain features such as chain length, unsaturation, and cyclopropane modification, collectively defining membrane states that modulate antibiotic susceptibility and tolerance phenotypes [7,8,9]. For example, *Vibrio alginolyticus* suppresses unsaturated fatty acids and phosphatidylethanolamine (PE) biosynthesis to diminish antibiotic efficacy, while perturbing phospholipid synthesis can reverse *mcr-1*-mediated polymyxin resistance in *Escherichia coli* [10,11]. Despite growing recognition of these structure–function links, routine lipidomics often lacks the structural resolution and annotation traceability required to connect subtle molecular species changes to physiological outcomes, particularly in bacteria with frequent coexisting isobaric and isomeric species. Therefore, elucidating the structural diversity and dynamic modifications of bacterial phospholipids is critical for deciphering antimicrobial resistance mechanisms and guiding targeted therapy development.

Current phospholipid analysis relies primarily on chromatographic separations and mass spectrometry (MS) techniques. Conventional chromatographic methods such as thin-layer chromatography (TLC) [12] and high-performance liquid chromatography (HPLC) [13] can efficiently separate phospholipid headgroup classes but provide limited information on acyl-chain composition and structural configurations. Consequently, MS has become central to lipidomic analysis due to its high sensitivity and molecular specificity [14,15]. However, unambiguous molecular species assignment remains challenging because multiple acyl-chain combinations and C=C positional isomers can explain the same precursor m/z, and many species share overlapping fragments. Chemical derivatization strategies such as the Paternò–Büchi reaction have been developed to address such limitations by allowing C=C localization through tandem MS [16,17,18]. Nevertheless, in the absence of routine integration, current pipelines still treat TLC, GC–MS, and LC–MS/MS as independent or parallel readouts, inflating the LC–MS/MS candidate space, limiting analytical efficiency, and increasing the risk that subtle remodeling signatures relevant to membrane physiology are missed or misassigned. Therefore, an integrated and orthogonal strategy enabling comprehensive, structure-resolved phospholipid profiles is needed, particularly for complex bacterial lipidomes where subtle structural variation produces important functional consequences.

**Figure 1:**
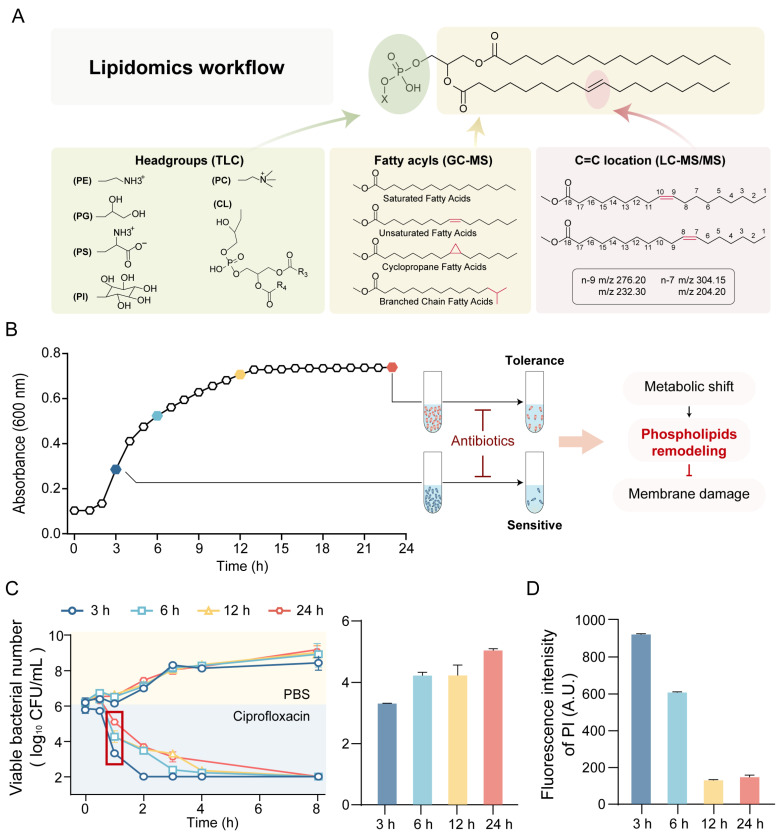
Membrane remodeling is associated with antibiotic tolerance in *E. coli*. (**A**) Schematic workflow of the analytical strategy for phospholipid structure characterization. (**B**) The growth curve of *E. coli* MG1655. The scheme on the right illustrates a hypothetical model associated with dynamic antibiotic tolerance. (**C**) Time-killing assays of *E. coli* collected at 3, 6, 12, and 24 h and treated with 0.0625 µg/mL ciprofloxacin (left). Differences in surviving bacterial numbers across growth phases at 1 h post-treatment (right). (**D**) Membrane permeability of *E. coli* across growth phases, determined by fluorescence intensity of propidium iodide. Data represent mean ± SD (*n* = 3).

Here, we established a constraint-driven phospholipid profiling workflow tailored for bacteria that integrates TLC-defined headgroup identity, GC–MS acyl-pool features, and PB-enabled C=C localization constraints to enumerate condition-specific candidate structures prior to LC–MS/MS confirmation (Figure 1A). Using *E. coli* as a model, we generated a molecular-species-resolved phospholipid atlas and quantified time-resolved remodeling of phospholipids. We further profiled remodeling signatures under sub-inhibitory antibiotic perturbations. To test the applicability of the workflow, we further extended the analysis to *Enterococcus faecium* strains, with or without vancomycin resistance genes. This study provides a structure-resolved and cross-validated view of bacterial phospholipid remodeling that supports biologically informed interpretation of membrane adaptation.

## 2. Results

### 2.1. Growth-Stage-Dependent Antibiotic Tolerance Model Established for Membrane Lipid Remodeling Analysis

Compared with log-phase bacteria, stationary-phase bacteria exhibit increased tolerance to antibiotics [19,20]. To examine the relationship between growth-stage-associated antibiotics tolerance and membrane phospholipid remodeling, *E. coli* cultures were sampled at 3, 6, 12, and 24 h to represent distinct growth phases (Figure 1B). Time-killing assays demonstrated pronounced stage-dependent differences in early survival following exposure to ciprofloxacin or colistin, with stationary-phase populations showing approximately tenfold higher viable counts at initial time points (Figure 1C and Figure S1A). In contrast, the growth rate and minimum inhibitory concentration (MIC) remained largely unchanged across growth stages (Figure S1B,C), indicating that the observed differences in survival were not attributable to altered intrinsic susceptibility or target-level resistance. Consistently, membrane permeability was significantly reduced in stationary phase bacteria (Figure 1D). Taken together, these results support an association between growth-phase-dependent antibiotic tolerance and membrane remodeling, thereby establishing a controlled framework for subsequent phospholipid compositional analysis and methodological validation.

### 2.2. CL Enrichment in Stationary-Phase E. coli Revealed by TLC

**Figure 2:**
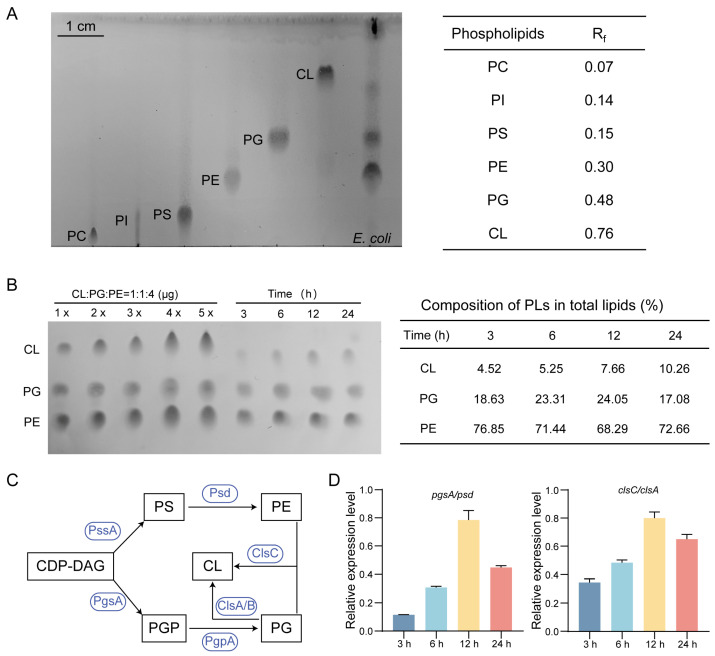
Dynamic changes in phospholipid headgroup composition in *E. coli*. (**A**) Phospholipid species are separated by TLC based on polarity and identified using authentic standards. PC: phosphatidylcholine; PI: phosphatidylinositol; PS: phosphatidylserine; PE: phosphatidylethanolamine; PG: phosphatidylglycerol; CL: cardiolipin. (**B**) TLC separation of PE, PG and CL using 1× – 5× standards to define the linear range for densitometry (left). Composition of major phospholipid classes at 3, 6, 12, and 24 h (right). (**C**) Scheme of the phospholipid biosynthetic pathway in *E. coli*. Corresponding synthases are shown in blue. (**D**) Relative expression levels of key phospholipid synthesis genes at different growth stages. Data represent mean ± SD (*n* = 3).

To define the phospholipid composition remodeling during growth, total phospholipids were first resolved by one-dimensional TLC. Authentic standards were used to assign phospholipid classes according to their TLC Rf values (Figure 2A), and densitometric analysis was further applied to estimate changes in the relative abundance of major phospholipid classes across growth stages (Figure 2B). At all-time points, PE, PG, and CL constituted the dominant phospholipid classes in *E. coli*, with a redistribution among these classes as cells approached the stationary phase (Figure 2B). PE remained the major component, declining from ~77% at 3 h to ~68% at 12 h before partially recovering at 24 h. In contrast, PG increased during exponential growth (~19% to ~24%) and subsequently decreased upon entry into the stationary phase (~17%). CL displayed the opposite trend, progressively accumulating from ~4.5% to ~10% by 24 h. Collectively, these results indicate a growth-stage-dependent redistribution among anionic phospholipids, with a transient enrichment of PG during exponential growth and increased CL abundance during the stationary phase. As an orthogonal class-level readout, these TLC results provided semi-quantitative support for major headgroup redistribution and informed subsequent molecular-species-resolved LC–MS/MS analysis.

Consistent with these compositional shifts, phospholipid biosynthetic genes exhibited coordinated growth-phase-dependent changes in transcript levels. The PG synthesis genes *pgsA* and *pgpA* were progressively upregulated throughout the logarithmic phase and reached high expression levels by 12 h (Figure S2A). PE synthesis genes showed a similar but gene-specific pattern, with *psd* peaking at 6 h and *pssA* reaching its maximum at 12 h before both returned toward baseline at 24 h (Figure S2B). Notably, CL synthase genes showed the most pronounced increase, as *clsA*, *clsB*, and *clsC* peaked at 12 h and decreased thereafter (Figure S2C). Given that CL synthesis utilizes PG as a substrate, and in the case of ClsC, can use PE as a phosphatidyl donor, the strong upregulation of *clsC/clsA* provides a plausible biosynthetic basis for the observed redistribution from PE and PG toward CL as cells approach the stationary phase (Figure 2D).

### 2.3. Suppression of Unsaturated Fatty Acid and Accumulation of Cyclopropane Species Across Growth Stages Demonstrated by GC–MS and PB–MS

**Figure 3:**
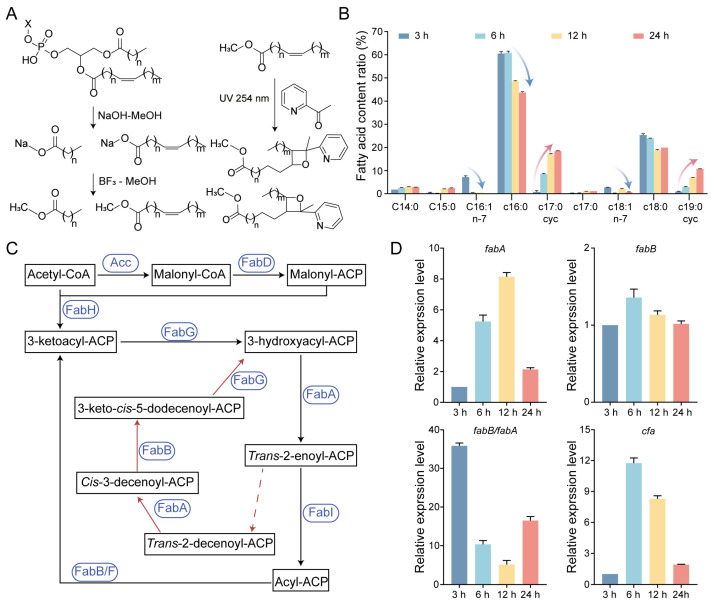
Dynamic changes in phospholipid acyl-chain composition in *E. coli*. (**A**) The workflow for fatty acyl methyl ester preparation (GC–MS) and Paternò–Büchi derivatization (LC–MS/MS) for C=C localization. (**B**) Phospholipid acyl chain composition across growth stages determined by GC–MS. Arrows indicate the major decrease in unsaturated fatty acids and the reciprocal increase in cyclopropane fatty acids during growth. (**C**) Scheme of the fatty acid biosynthetic pathway in *E. coli*, with corresponding synthases shown in blue. Black arrows indicate the saturated fatty acid (SFA) synthesis pathway, whereas red arrows indicate the unsaturated fatty acid (UFA) synthesis pathway. Dashed arrows indicate possible branch points at which intermediates may enter the UFA cycle, rather than direct enzymatic reactions. (**D**) Relative expression levels of key fatty acid synthesis genes at different growth stages. Data represent mean ± SD (*n* = 3).

To further refine phospholipid structural annotation, we profiled phospholipid acyl chains via GC–MS, and C=C positional isomers were resolved using Paternò–Büchi derivatization coupled to LC–MS/MS (Figure 3A) [21,22]. The fatty acyl pool comprised C14:0, C15:0, C16:1 n-7, C16:0, C17:0 (cyc), C17:0, C18:1 n-7, C18:0 and C19:0 (cyc), with C16:0 as the dominant component across growth stages (Figure 3B and Figure S3). In contrast, the relative abundance of unsaturated fatty acids decreased markedly (~10% to <1%) over time, whereas cyclopropane species progressively accumulated to ~29% by 24 h, consistent with cyclopropanation of pre-existing unsaturated acyl chains [23]. The paired decrease in C16:1 n-7 and C18:1 n-7, together with the corresponding increase in C17:0 (cyc) and C19:0 (cyc), further supports the conversion of pre-existing unsaturated acyl chains into cyclopropane derivatives. To assess the relative capacity for de novo unsaturated fatty-acid synthesis, we examined the *fabB/fabA* expression ratio, as FabA and FabB jointly establish and elongate the unsaturated acyl ACP branch in *E. coli* (Figure 3C). The reduced *fabB/fabA* ratio from 6 to 24 h is consistent with diminished unsaturated acyl-chain supply. Together with increased *cfa* expression, this reduced unsaturated acyl-chain supply could contribute to the observed depletion of C16:1 and C18:1 and the accumulation of cyclopropane species during late growth (Figure 3D). Together, these acyl-level constraints, combined with PB-defined C=C localization, provide a traceable framework that narrows the LC–MS/MS candidate space by restricting permissible acyl combinations and double-bond positions in subsequent molecular species annotation.

### 2.4. Rule-Based Ion Prediction Narrows LC–MS/MS Candidate Space

**Figure 4:**
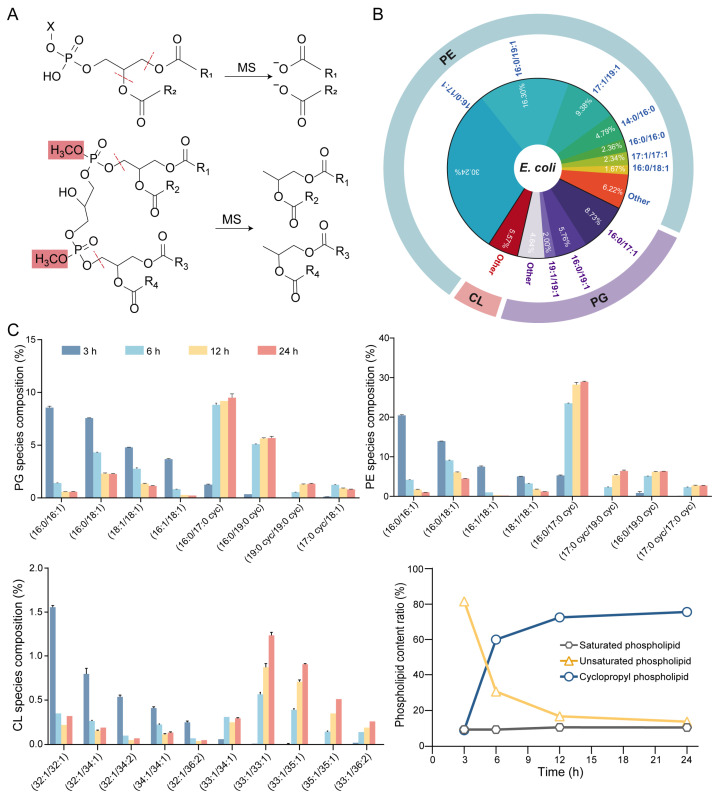
Dynamic changes in phospholipid structures of *E. coli*. (**A**) Fragmentation patterns of phospholipid molecules in MS. PE and PG are analyzed in negative ion mode, where the deprotonated precursor ions [M − H]^−^ produce characteristic acyl-chain fragments. CL is methylated and analyzed in positive ion mode as ammonium adducts [M + NH_4_]^+^. (**B**) The composition of major phospholipid species of *E. coli* at 12 h. (**C**) Dynamic changes in structures of PE, PG, and CL molecules at 3, 6, 12, and 24 h. The lower right panel summarizes the overall proportions of saturated, unsaturated, and cyclopropyl-containing phospholipids during different growth phases. Data represent mean ± SD (*n* = 3).

Using TLC-defined headgroup classes together with GC–MS- and PB–MS-derived acyl features, we enumerated condition-specific candidate phospholipid structures and constrained the LC–MS/MS search space according to the principle of orthogonal structural constraints in lipidomics [24]. We then analyzed a panel of phospholipid standards to define class-preferred adduct forms and fragmentation behaviors under our acquisition settings and encoded these behaviors into an in-house ion prediction table that serves as a rule-based reference for LC–MS/MS method development under comparable analytical settings (Figure 4A, Table 1) [25]. For each lipid class, this ion prediction table specifies the preferred ionization/adduct form and calculates (i) the exact precursor m/z and (ii) class and acyl-chain diagnostic product ions, enabling rapid generation of transition lists for targeted assays (MRM/PRM) across LC–MS/MS platforms. Because the predicted ions are derived from exact-mass formulas and class-specific fragmentation logic, the library can serve as a reference for transition design on other LC–MS/MS platforms, although instrument-specific optimization is still required. Within this workflow, PG, phosphatidylserine (PS), phosphatidylinositol (PI), PE, and phosphatidylcholine (PC) were primarily detected in negative ion mode as [M + HCOO]^−^ or [M − H]^−^. CL, a low-abundance dimeric phospholipid in bacteria, was methylated to improve detectability and measured in positive ion mode as [M + NH_4_]^+^ [15]. Collectively, this strategy integrates orthogonal structural constraints with rule-based ion prediction to enhance annotation confidence and reproducibility while substantially reducing combinatorial ambiguity in LC–MS/MS-based phospholipid analysis.

### 2.5. Molecular Species Analysis of CL Accumulation and Cyclopropanation at Stationary Phase

Using this platform, we established a molecular-species-resolved phospholipid atlas of *E. coli* at mid-growth phase (12 h). We defined the dominant PG, PE, and CL species and provided a reference baseline for subsequent dynamic analyses (Figure 4B, Table S2). The major species were largely built from a limited set of recurring acyl features, most commonly pairing a saturated C16:0 chain with either an unsaturated C16:1 n-7 chain or a cyclopropane C17:0 (cyc) chain. Consistent with CL biosynthesis drawing on PG (and PE as a phosphatidyl donor via ClsC), the prevalent CL species shared the dominant acyl features observed in PG and PE. We further quantified time-resolved remodeling across 3, 6, 12, and 24 h at the molecular species level (Figure 4C, Table S3). Although the total fraction of fully saturated phospholipids remained near 10% throughout growth, the unsaturated fraction rapidly decreased from >80% at 3 h to single-digit percentages by late growth, accompanied by a marked accumulation of cyclopropane-containing species (Figure 4C, lower right). At the species level, this shift was reflected by a coordinated decreases in C16:1 n-7-containing PG and PE species and reciprocal increases in the corresponding C17:0 (cyc)-containing species, in agreement with the GC–MS results. In addition, the class-level trends derived from LC–MS/MS were consistent with the corresponding TLC-based semi-quantitative estimates of PE, PG, and CL across growth stages (Table S4), despite modest differences in absolute percentages between methods. Together, these data define a late-growth membrane state characterized by increased CL abundance, reduced unsaturation, and enhanced cyclopropanation. Notably, the concordance among TLC, GC–MS, and LC–MS/MS highlights the value of orthogonal constraints for cross-validating phospholipid remodeling patterns and strengthening confidence in molecular-species-resolved annotation.

### 2.6. Phospholipid Remodeling Under Antibiotic Perturbations and Resistance

**Figure 5:**
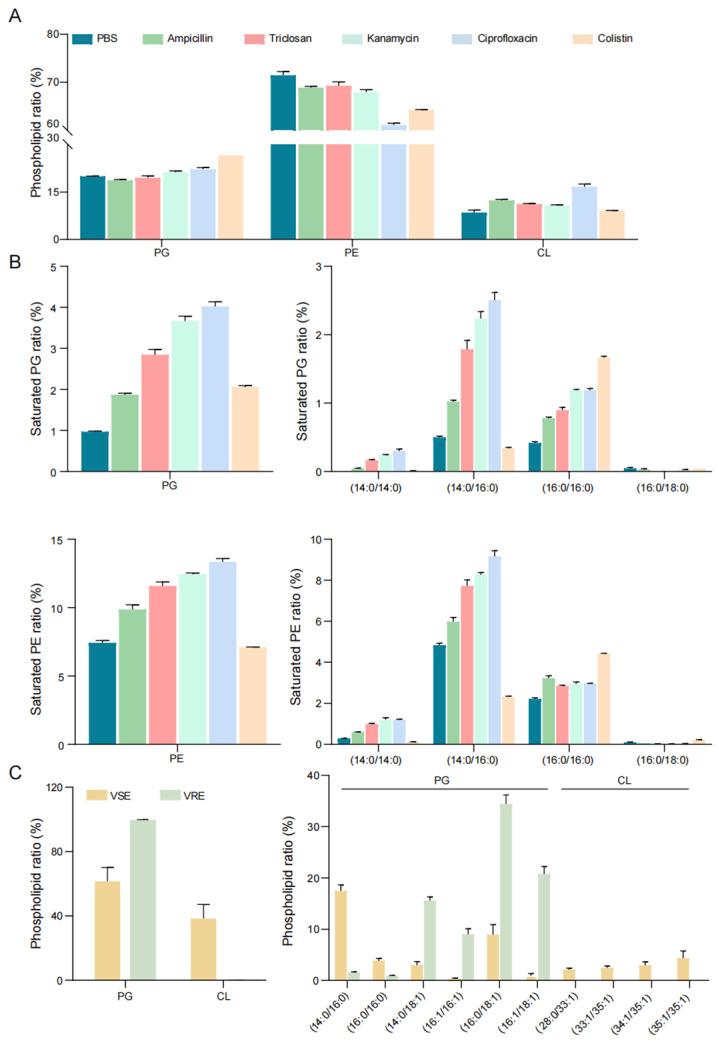
Antibiotic perturbations and resistance remodel bacterial phospholipids. (**A**) *E. coli* was cultured for 24 h with sub-inhibitory antibiotics (0.2 × MIC; ampicillin, triclosan, kanamycin, ciprofloxacin, or colistin) or the PBS control, followed by phospholipid profiling. Relative abundances of major phospholipid classes (PG, PE, and CL) are shown. (**B**) Antibiotic exposure increases saturated phospholipids fractions. Left, total fraction of saturated PG and saturated PE. Right, relative abundances of major saturated PG and PE molecular species (acyl-chain composition indicated). (**C**) Phospholipid remodeling in *Enterococcus faecium* is associated with vancomycin resistance. Relative abundances of PG and CL in vancomycin-sensitive *E. faecium* (VSE) and vancomycin-resistant *E. faecium* (VRE) strains (left). Relative abundances of representative PG and CL molecular species (right). Data represent mean ± SD (*n* = 3).

To examine phospholipid remodeling under antibiotic stress, *E. coli* cultures were grown for 24 h in the presence of sub-inhibitory concentrations (0.2× MIC) of antibiotics targeting distinct cellular processes. The phospholipids were analyzed using the same constraint-driven workflow (Table S5). Compared with the control, antibiotic-exposed cultures showed increased PG and CL fractions, with a concomitant decrease in PE (Figure 5A). Molecular species analysis further revealed an increased abundance of saturated PG and PE species (Figure 5B). The saturated PE fraction increased despite an overall decrease in total PE, indicating that sub-inhibitory antibiotic exposure selectively enriches higher-saturation PE species within a shrinking PE pool. Notably, under low antibiotic concentrations, we also detected higher proportions of short-chain phospholipids, particularly (14:0/14:0) and (14:0/16:0) species. This compositional shift reflects preferential incorporation of shorter acyl precursors under antibiotic stress, although the underlying biosynthetic basis remains to be established.

To test the workflow in an additional bacterial system with a distinct phospholipid background, we next compared phospholipid profiles of vancomycin-sensitive and vancomycin-resistant strains of the Gram-positive pathogen *E. faecium* (Table S6). Although *E. faecium* membranes also contain neutral lipids and glycolipids, the present analysis was intentionally restricted to the dominant phospholipid classes PG and CL to provide a phospholipid-focused comparison. In vancomycin-sensitive *E. faecium* (VSE), PG and CL accounted for approximately 60% and 40% of total phospholipids, respectively, whereas vancomycin-resistant *E. faecium* (VRE) exhibited a marked shift toward PG dominance (>90%, Figure 5C). At the molecular species level, VSE strains were characterized by fully saturated PG species such as PG (30:0–14:0/16:0) and PG (32:0–16:0/16:0). By contrast, VRE strains showed enrichment of unsaturated PG species, including PG (34:1–16:0/18:1) and PG (34:2–16:1/18:1) (Figure 5C, right). Together, these findings demonstrate that the platform enables rapid, class- and molecular-species-resolved phospholipid quantification with traceable annotation across antibiotic perturbations and in an additional Gram-positive model.

## 3. Discussion

Bacterial phospholipids are essential determinants of membrane organization, permeability, and protein functionality. Dynamic phospholipid remodeling represents a central adaptive response to environmental and chemical stressors, with bacteria modifying phospholipid composition during growth-phase transitions or under antibiotic challenge to maintain membrane integrity and homeostasis [26,27,28,29]. Here, we developed an integrated phospholipid profiling platform that combines orthogonal analytical constraints with class-specific diagnostic MS/MS ions to enable molecular-species-resolved, traceable annotation. Application of this framework to both growth-stage transitions and sub-inhibitory antibiotic exposure revealed a convergent remodeling trajectory characterized by CL enrichment, reduced unsaturation, and enhanced cyclopropanation. In contrast, VRE displayed a distinct remodeling pattern with near-complete PG dominance and enrichment of unsaturated PG molecular species, underscoring that phospholipid remodeling strategies can diverge markedly across species and resistance contexts.

Bacterial lipidomes are highly species-specific in both headgroup composition and acyl-chain architecture [7,30,31]. Accordingly, MS and MS/MS are core tools for high-throughput phospholipid profiling [32]. Recent studies have developed automated MS/MS-driven pipelines for phospholipid identification. For instance, LipidHunter enables high-throughput identification by matching collision-induced dissociation (CID) product ions and neutral-loss features against class-specific rules and user-defined fatty-acyl whitelists, thereby distinguishing lipids with the same nominal mass but different classes or fatty-acyl compositions [33]. However, similar to other MS/MS-only pipelines, confident molecular-species assignment can remain sensitive to the choice of whitelist entries and the size of the candidate space in complex bacterial lipidomes. In this study, beyond defining headgroup classes and acyl-chain compositions, we introduce the Paternò–Büchi reaction, which additionally provides C=C information and captures acyl-chain modifications such as cyclopropanation. For example, in both the growth phase model and the antibiotic exposure model, we observed a coordinated decrease in C16:1-containing phospholipid species with a reciprocal enrichment of the corresponding C17:0 cyclopropane-containing species, consistent with the acyl-pool trends measured by GC–MS, highlighting the ability of the platform to resolve dynamic membrane remodeling at the molecular species level. Altogether, our workflow introduces orthogonal structural constraints that add more information beyond headgroup classes and acyl-chain composition to predefine plausible molecular-species hypotheses, reduce the LC–MS/MS candidate space, and improve annotation traceability.

Phospholipid remodeling involves coordinated changes in both acyl-chain structure and headgroup composition. Cyclopropane fatty acids represent a conserved bacterial strategy to stabilize membrane structure under stress [34]. Previous work has shown that *Latilactobacillus sakei* carrying *cfa* upregulates C19:0 cyclopropane under high CO_2_ to reduce membrane permeability and enhance acid tolerance [35]. Similarly, deletion of *cfa* in *Salmonella typhimurium* compromises survival under acidic and oxidative conditions, supporting a role for cyclopropane formation in long-term persistence and resistance to oxidative damage [36]. In line with these reports, our data resolve a molecular-species shift from C16:1-containing phospholipids toward C17:0 cyclopropane-containing species accompanied by decreased membrane permeability, consistent with cyclopropanation contributing to a barrier-strengthened membrane state under late-growth or antibiotic-associated stress. We also detected CL enrichment, increasing from approximately 5% to more than 10%. Prior work reported that high-salt or high-osmotic-pressure conditions induce *E. coli* to express *cls* and increase CL content while decreasing PE and shifting the membrane toward a higher anionic-lipid fraction [37,38]. At the same time, CL is enriched and tends to aggregate at highly curved cell poles and division septa, co-localizing with many functional proteins such as ProP and MurG, suggesting that CL may be preferentially retained or synthesized under stress and may contribute to membrane organization compatible with protein function [37,39]. Together with the cyclopropanation-shift signature and reduced permeability, these results support the co-occurrence of cyclopropanation and CL enrichment as components of a late-growth or stress-associated remodeling trajectory, in which cyclopropanation strengthens barrier properties while CL contributes to curvature-dependent organization and protein–lipid interactions.

Our findings further show that the workflow can be applied to a Gram-positive model, *E. faecium*, in which a distinct remodeling trajectory was observed. Compared with Gram-positive pathogens such as *S. aureus*, *E. faecium* naturally contain a relatively high proportion of CL, which may reflect adaptation to acidic or stress-associated ecological niches [30]. However, acquisition of the *vanA* plasmid was accompanied by a marked shift toward PG dominance, yielding a distinct PG-enriched phenotype in resistant strains. This remodeling pattern contrasts with the CL accumulation observed in *E. coli*, suggesting that envelope reconfiguration in *vanA*-mediated resistance follows a different structural logic. Although direct mechanistic links remain to be established, *vanA*-dependent alterations in peptidoglycan cross-linking and surface charge may secondarily influence membrane–cell wall coupling and lipid distribution [40,41]. Overall, orthogonal constraint integration enables cross-validated, species-resolved phospholipid annotation, and sensitive detection of remodeling trajectories in bacteria.

Despite these insights, several limitations of the present study should be noted. First, although the current data reveal robust associations between phospholipid remodeling and antibiotic tolerance, the direct causality has not been clarified. Targeted genetic and functional perturbation will be required in future work to determine whether specific phospholipid remodeling events directly contribute to antibiotic tolerance. Second, the present workflow provides a molecular-species-resolved phospholipid profile under the current analytical conditions, but cardiolipin analysis relies on methylation-assisted detection. Although this strategy improves CL detectability, it may still introduce derivatization-related bias or differential recovery, particularly for low-abundance species, and may partly contribute to the differences in class-level CL proportions observed between TLC and LC–MS/MS. Third, although the platform provides detailed bulk compositional data, it does not resolve leaflet asymmetry, localization between membrane sub-compartments, or membrane microdomain organization. Therefore, the observed association with reduced membrane permeability should not be interpreted as direct structural proof of these spatial membrane features. In future work, integration with targeted genetic validation, improved class-specific quantification strategies, and spatially resolved lipidomics or phospholipid-targeted fluorescence imaging will help further clarify the mechanistic and spatial basis of membrane remodeling during antibiotic adaptation.

## 4. Materials and Methods

### 4.1. Bacterial Strains and Culture Conditions

*Escherichia coli* MG1655, *Enterococcus faecium* BM4105-RF, and *Enterococcus faecium* BM4105 (*vanA*) were stored as glycerol stocks (25%, *v*/*v*) at −80 °C. Unless otherwise stated, a single colony was inoculated into brain–heart infusion (BHI, Beijing Land Bridge Technology, Beijing, China) broth and cultured at 37 °C under shaking at 200 revolutions per minute (r.p.m.).

### 4.2. Chemicals and Reagents

PG (34:1-16:0/18:1), PE (34:1-16:0/18:1), PC (34:1-16:0/18:1), PI (34:1-16:0/18:1), PS (34:1-16:0/18:1), PA (16:0/18:1), and CL (56:4-14:1/14:1/14:1/14:1) were purchased from Merck (Beijing, China). Boron trifluoride in methanol solution, n-hexane, methyl tert-butyl ether, trimethylsilyl diazomethane, and fatty acid methyl ester (FAME) mixture standards were purchased from Sigma-Aldrich (Beijing, China). Isopropanol, chloroform, and methanol (MS grade) were purchased from Fisher Scientific (Fair Lawn, NJ, USA).

### 4.3. Growth Curves

Overnight cultures were adjusted to a 0.5 McFarland turbidity standard, diluted 1:100 in lysogeny broth (LB, Beijing Land Bridge Technology), and dispensed as 200 μL into 96-well microplates with lids. Growth was monitored by measuring OD_600_ every 1 h at 37 °C using an Infinite M200 microplate reader (Tecan Group Ltd., Männedorf, Switzerland) in kinetic mode. Based on the growth curves, the subsequent bacterial samples were collected at 3, 6, 12, and 24 h to represent distinct growth stages.

### 4.4. Antibacterial-Susceptibility Test

Minimum inhibitory concentrations (MICs) were determined by the broth microdilution method. Briefly, twofold serial dilutions of antibiotics were prepared in Mueller–Hinton II (MHII) broth in 96-well microtiter plates and mixed with an equal volume of bacterial suspension in MHII to achieve a final inoculum of approximately 5 × 10^5^ CFU/mL. Plates were incubated at 37 °C for 18 h, and the MICs were defined as the lowest antibiotic concentration showing no visible bacterial growth.

### 4.5. Time-Dependent Killing Assay

Cultures collected at 3, 6, 12, and 24 h were diluted in LB to a final concentration of 1 × 10^6^ CFU/mL. Ciprofloxacin or colistin (4 × MIC) was added, and cultures were incubated at 37 °C under shaking at 200 r.p.m. At 0.5, 1, 2, 3, 4, and 8 h post-treatment, 100 μL aliquots were removed, serially diluted tenfold, and plated on Mueller–Hinton agar plates for viable counting. Colonies were enumerated after incubation at 37 °C for 24 h, and viable counts were expressed as CFU/mL.

### 4.6. Membrane Integrity Assays

Bacterial samples were centrifuged and resuspended in PBS to an OD_600_ of 0.5. Propidium iodide (PI) was added to a final concentration of 10 μM. Suspensions were incubated at 37 °C for 20 min while protected from light, and fluorescence was measured at an excitation/emission wavelength of 535/615 nm.

### 4.7. Total Lipid Extraction

Lipids were extracted using a modified Bligh & Dyer method. Briefly, bacterial suspensions were washed twice with PBS. Cell pellets were collected by centrifugation at 4500 r.p.m. for 15 min, and the wet pellet mass was recorded. Pellets were resuspended in PBS and extracted with chloroform/methanol (1:2, *v*/*v*) using a solvent volume threefold that of PBS. Samples were vortexed and incubated at 4 °C for 90 min. The mixture was centrifuged at 3000 r.p.m. for 10 min. The lower organic phase (chloroform layer) was collected and dried under a gentle stream of nitrogen. Dried lipids were re-dissolved in chloroform/methanol (2:1, *v*/*v*) to 1 μL/mg and stored at −80 °C for subsequent analysis.

### 4.8. Analysis of Phospholipid Head Groups in Pathogens

Phospholipid composition was analyzed by thin-layer chromatography (TLC). Briefly, phospholipid extracts were spotted onto silica gel TLC plates (Merck KGaA, Darmstadt, Germany). The TLC plate and developing chamber were pre-saturated with chloroform/methanol/acetic acid (65:25:10, *v*/*v*/*v*) for 1 h, and the plate was developed for 15 min. After drying, the plate was sprayed with 10% phosphomolybdate reagent for 1 min and then heated at 145 °C for visualization.

### 4.9. Phospholipid Acyl-Chain Composition Analysis

The phospholipid acyl chains were converted to fatty acid methyl esters (FAMEs) and analyzed by GC–MS. Phospholipid samples were saponified with 2 mL of 10 mM NaOH–methanol solution at 65 °C for 30 min, followed by esterification with 1 mL of BF_3_-methanol at 100 °C for 10 min. The resulting FAMEs were extracted with 2 mL hexane and washed with 2 mL saturated NaCl solution. Samples were centrifuged at 3000× *g* for 5 min, and the upper organic phase was collected. The aqueous phase was re-extracted with 2 mL hexane, centrifuged, and the upper organic phase was collected. Combined organic phases were evaporated under nitrogen.

Dried FAMEs were re-dissolved in hexane and analyzed using a Shimadzu GCMS-QP2020NX system (Shimadzu Corporation, Kyoto, Japan) equipped with an SH-I-5Sil capillary column (30 m × 0.25 mm × 0.25 μm; Shimadzu Corporation, Kyoto, Japan). Helium was used as the carrier gas at 1 mL/min. The injector temperature was set to 225 °C. The GC oven program was as follows: 70 °C for 1 min, ramp to 160 °C at 20 °C/min and hold for 5 min, then ramp to 225 °C at 2 °C/min and hold for 30 min. A local volatile-compound library was used for peak annotation. Relative fatty acid abundance was calculated based on peak area, without correction factors.

To analyze the unsaturated bond position, dried FAMEs were re-dissolved in 1 mL acetonitrile containing 30 mmol/L of 2-acetylpyridine. After irradiation under a 254 nm mercury lamp for 20 min, samples were analyzed by LC–MS/MS.

### 4.10. Phospholipid Profiling Assay

For CL methylation, phospholipid extracts were reacted with 50 μL (trimethylsilyl) diazomethane (TMSD; 2 M in hexane) in 400 μL methyl tert-butyl ether/methanol (20:6, *v*/*v*) for 20 min at room temperature. Excess TMSD was quenched by adding 5 μL glacial acetic acid, followed by the addition of 92 μL water. Samples were centrifuged at 12,000× *g* for 20 min to collect methylated CL in the organic phase. Methylated CL and phospholipid samples were combined and then re-dissolved in isopropanol/acetonitrile (9:1, *v*/*v*) for LC–MS/MS analysis. All samples were subjected to identical derivatization conditions and analyzed within the same workflow to minimize systematic bias across groups. In addition, derivatized standards were processed in parallel as a procedural control to monitor derivatization performance under the applied conditions.

Phospholipid classes and acyl-chain characteristics were analyzed using a LC–MS 8045 system (Shimadzu, Kyoto, Japan). Chromatographic separation was performed on a C8 column (150 × 2.1 mm, 2.6 μm; Phenomenex, Torrance, CA, USA). Mobile phase A was isopropanol/acetonitrile (90:10, *v*/*v*) containing 0.1% formic acid and 10 mM ammonium formate, and mobile phase B was acetonitrile/water (60:40, *v*/*v*) containing 0.1% formic acid and 10 mM ammonium formate. The gradient program was as follows: 0 min, 60% B; 3 min, 60% B; 23 min, 2% B; 30 min, 2% B; 30.5 min, 60% B; 35 min, 60% B. The flow rate was 0.3 mL/min. Lipid extracts were analyzed in scheduled MRM mode in negative ionization for Lyso-PG, Lyso-PE, Lyso-PC, PE, PS, PA, PI, PG, and PC and in positive ionization for CL.

### 4.11. qRT-PCR Assay

Bacterial total RNA was extracted using an RNA Mini Kit (Magen Biotechnology, Guangzhou, China) following the manufacturer’s protocol. The concentration and purity of total RNA was monitored using a NanoDrop 2000 spectrophotometer (Thermo Fisher Scientific, Waltham, MA, USA). A total of 500 ng RNA was reverse transcribed into cDNA using a reverse transcription kit (Takara, Dalian, China). Transcript levels of *pgsA*, *pgpA*, *psd*, *pssA*, *clsA*, *clsB*, *clsC*, *fabA*, *fabB*, and *cfa* were quantified using a QuantStudio™ 7 Flex Real-Time PCR System (Applied Biosystems, Waltham, MA, USA) with SYBR Green Master Mix (Thermo Fisher Scientific, Waltham, MA, USA) and primers listed in Table S1. Relative expression was calculated using the 2^−ΔΔCt^ method, with 16S rRNA as an endogenous control. All samples were analyzed in triplicate.

## 5. Conclusions

In this study, we established a constraint-driven phospholipid profiling platform that integrates orthogonal structural evidence with MS/MS diagnostic ion prediction to achieve high-confidence, molecular-species-resolved annotation in bacterial lipidomics. By substantially reducing structural ambiguity, this framework enables systematic and comparable analysis of membrane remodeling across growth conditions, antibiotic perturbations, and multiple bacterial models. Our findings further define a membrane state associated with antibiotic tolerance, characterized by CL enrichment and reduced unsaturation, and provide compositional insight into lipid-mediated adaptation. Collectively, our work establishes a scalable foundation for future mechanistic and causal investigations of lipid remodeling and highlights membrane lipid homeostasis as a promising area for antimicrobial target discovery.

## Figures and Tables

**Table 1:** Rule-based ion prediction for phospholipid precursors and featured fragment m/z values for LC–MS/MS annotation and targeted method development.

Phospholipid	Polarity	Precursors	Featured Fragment 1	Featured Fragment 2
Lyso-PG	Negative (−H)	12 × (a + 6) + 1.0078 × (2a − 2m + 12) + 15.9949 × 9 + 30.9738	12 × a + 1.0078 × (2a − 2m) + 15.9949 × 2 − 1.0078	
Lyso-PE	Negative (−H)	12 × (a + 5) + 1.0078 × (2a − 2m + 11) + 15.9949 × 7 + 30.9738 + 14.0031	12 × a + 1.0078 × (2a − 2m) + 15.9949 × 2 − 1.0078	
Lyso-PC	Negative (−H)	12 × (a + 8) + 1.0078 × (2a − 2m + 17) + 15.9949 × 7 + 30.9738 + 14.0031	12 × a + 1.0078 × (2a − 2m) + 15.9949 × 2 − 1.0078	
PA	Negative (−H)	12 × (a + b + 3) + 1.0078 × (2a + 2b − 2m − 2n + 4) + 15.9949 × 8 + 30.9738	12 × a + 1.0078 × (2a − 2m) + 15.9949 × 2 − 1.0078	12 × b + 1.0078 × (2b − 2n) + 15.9949 × 2 − 1.0078
PC	Negative (+HCOO)	12 × (a + b + 9) + 1.0078 × (2a + 2b − 2m − 2n + 17) + 15.9949 × 10 + 30.9738 + 14.0031	12 × a + 1.0078 × (2a − 2m) + 15.9949 × 2 − 1.0078	12 × b + 1.0078 × (2b − 2n) + 15.9949 × 2 − 1.0078
PS	Negative (−H)	12 × (a + b + 6) + 1.0078 × (2a + 2b − 2m − 2n + 9) + 15.9949 × 10 + 30.9738 + 14.0031	12 × a + 1.0078 × (2a − 2m) + 15.9949 × 2 − 1.0078	12 × b + 1.0078 × (2b − 2n) + 15.9949 × 2 − 1.0078
PI	Negative (−H)	12 × (a + b + 9) + 1.0078 × (2a + 2b − 2m − 2n + 14) + 15.9949 × 13 + 30.9738	12 × a + 1.0078 × (2a − 2m) + 15.9949 × 2 − 1.0078	12 × b + 1.0078 × (2b − 2n) + 15.9949 × 2 − 1.0078
PE	Negative (−H)	12 × (a + b + 5) + 1.0078 × (2a + 2b − 2m − 2n + 9) + 15.9949 × 8 + 30.9738 + 14.0031	12 × a + 1.0078 × (2a − 2m) + 15.9949 × 2 − 1.0078	12 × b + 1.0078 × (2b − 2n) + 15.9949 × 2 − 1.0078
PG	Negative (−H)	12 × (a + b + 6) + 1.0078 × (2a + 2b − 2m − 2n + 10) + 15.9949 × 10 + 30.9738	12 × a + 1.0078 × (2a − 2m) + 15.9949 × 2 − 1.0078	12 × b + 1.0078 × (2b − 2n) + 15.9949 × 2 − 1.0078
CL	Positive (+NH_4_)	12 × (a + b + c + d + 11) + 1.0078 × (2a + 2b + 2c + 2d − 2m − 2n − 2o − 2p + 22) + 15.9949 × 17 + 30.9738 × 2 + 14.0031	12 × (a + b + 3) + 1.0078 × (2a + 2b − 2m − 2n + 3) + 15.9949 × 4	12 × (c + d + 3) + 1.0078 × (2c + 2d − 2o − 2p + 3) + 15.9949 × 4

Note: a, b, c, and d denote the carbon numbers of acyl chains 1–4, and m, n, o, and p denote the numbers of C=C bonds in the corresponding chains. The equations yield exact precursor *m*/*z* values and featured fragments for constructing LC–MS/MS transition lists; platform-specific adaptation may require optimization of source conditions, collision settings, and other instrument-dependent acquisition parameters.

## Data Availability

All data are contained in the article and the accompanying supplemental data.

## Supplementary Materials

The following supporting information can be downloaded at: https://www.mdpi.com/article/10.3390/antibiotics15050459/s1, Figure S1: Dynamic antibiotic tolerance in *E. coli*.; Figure S2: Dynamic expression of phospholipid biosynthesis genes in *E. coli*.; Figure S3: GC–MS TIC chromatograms of FAMEs from total lipid extracts of *E. coli* collected at 3, 6, 12, and 24 h.; Table S1: RT–qPCR primers used in this study.; Table S2: Phospholipid ratios (%) of *E. coli* at 12 h.; Table S3: Phospholipid ratios (%) of *E. coli* at different growth stages.; Table S4: Comparison of phospholipid class proportions determined by TLC densitometry and LC–MS/MS across growth stages in *E. coli*.; Table S5: Phospholipid ratios (%) of *E. coli* under sub-inhibitory antibiotics.; Table S6: Phospholipid ratios (%) of VSE and VRE.
