# Supplementary material for: Rule-Based Ion Prediction with Orthogonal Constraints Reveals Bacterial Phospholipid Remodeling Signatures
Source: Antibiotics (Basel). 2026 Apr 30;15(5):459. doi: 10.3390/antibiotics15050459 (PMC13203173; doi:10.3390/antibiotics15050459)
Supplement: Supplementary file 1 [file antibiotics-15-00459-s001.zip › antibiotics-4266799-supplementary.pdf]

**Supporting Information**

**Rule-based ion prediction with orthogonal constraints reveals bacterial hospholipid remodeling signatures**

Wanying Hu[1#], Wenhan Li[1#], Meirong Song[1], Jianfei Zhu[1], Kui Zhu[1, 2, 3*]

[1] State Key Laboratory of Veterinary Public Health and Safety, College of Veterinary Medicine, China Agricultural University, Beijing 100193, China.

[2] Engineering Research Center of Animal Innovative Drugs and Safety Evaluation, Ministry of Education, College of Veterinary Medicine, China Agricultural University, Beijing 100193, China.

[3] Technology Innovation Center for Food Safety Surveillance and Detection (Hainan), Sanya Institute of China Agricultural University, Sanya 572025, China.

[#]These authors contributed equally to this work.

[*]Corresponding author: Prof. Kui Zhu. Email: zhuk@cau.edu.cn.

## Content list

### Supplementary Figures

Supplementary Fig. 1----------------------------------------------------------------3

Supplementary Fig. 2----------------------------------------------------------------4

Supplementary Fig. 3----------------------------------------------------------------5

### Supplementary Tables

Supplementary Table 1-------------------------------------------------------------6

Supplementary Table 2-------------------------------------------------------------7

Supplementary Table 3-------------------------------------------------------------9

Supplementary Table 4-------------------------------------------------------------12

Supplementary Table 5-------------------------------------------------------------13

Supplementary Table 6-------------------------------------------------------------21

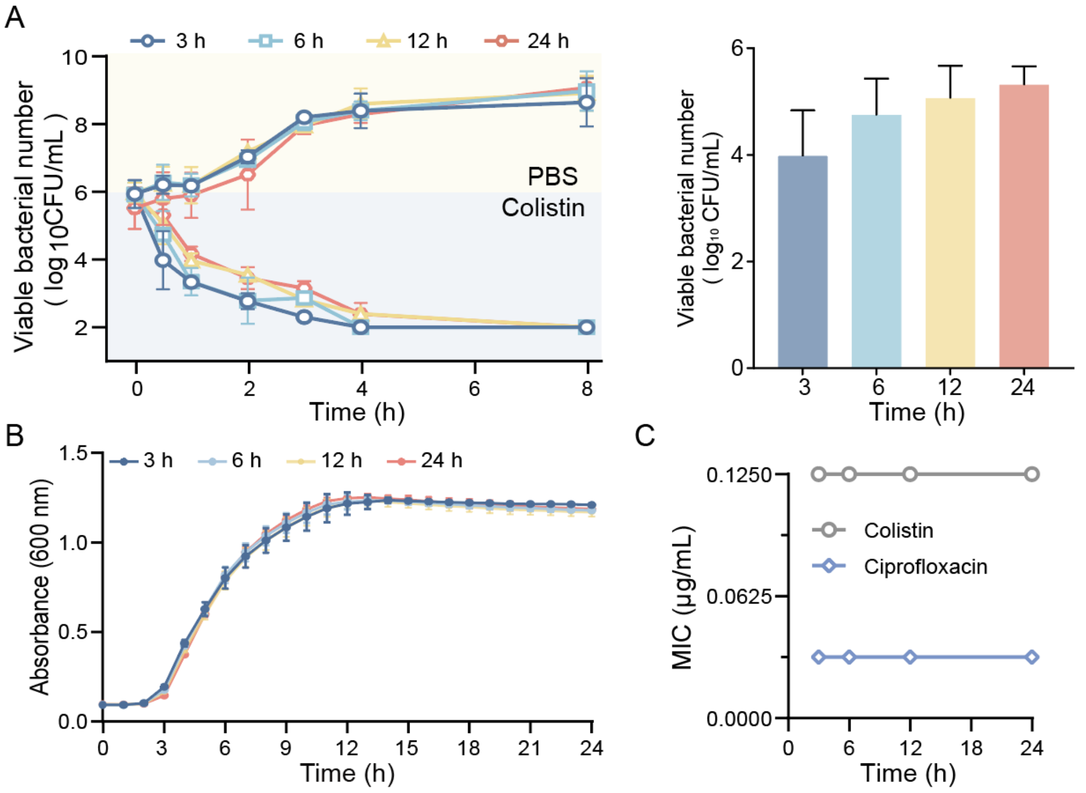


**Supplementary Figure S1. Dynamic antibiotic tolerance in *E. coli*.** (A) Time-kill assays of bacteria samples exposed to 4× MIC colistin. The right panel quantifies viable counts at 0.5 h post-treatment for each growth phase. (B) Growth curve of cultures harvested at 3, 6, 12, and 24 h after inoculation, representing distinct growth phases. (C) MIC values of colistin and ciprofloxacin determined for cultures harvested at the indicated time points.

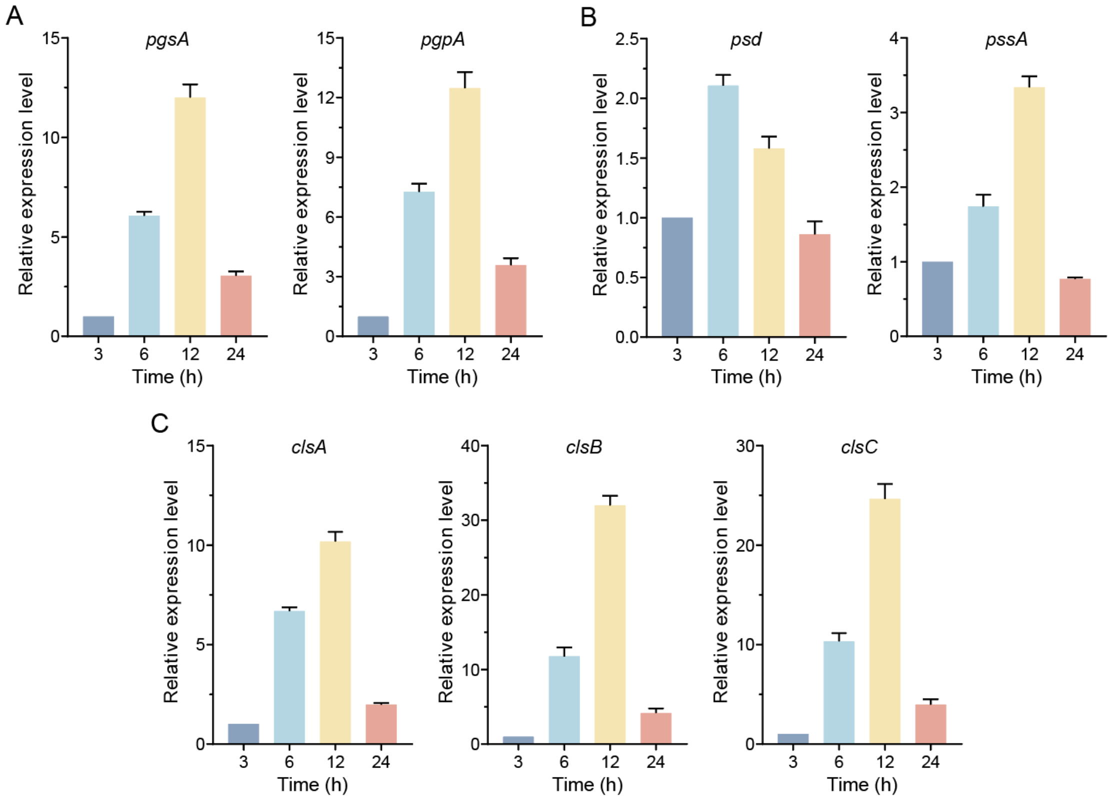


**Supplementary Figure S2. Dynamic expression of phospholipid biosynthesis genes in *E. coli*.** Relative transcript levels of phospholipid biosynthetic genes were quantified at 3, 6, 12, and 24 h. (A) PG pathway genes. (B) PE pathway genes. (C) CL synthase genes.

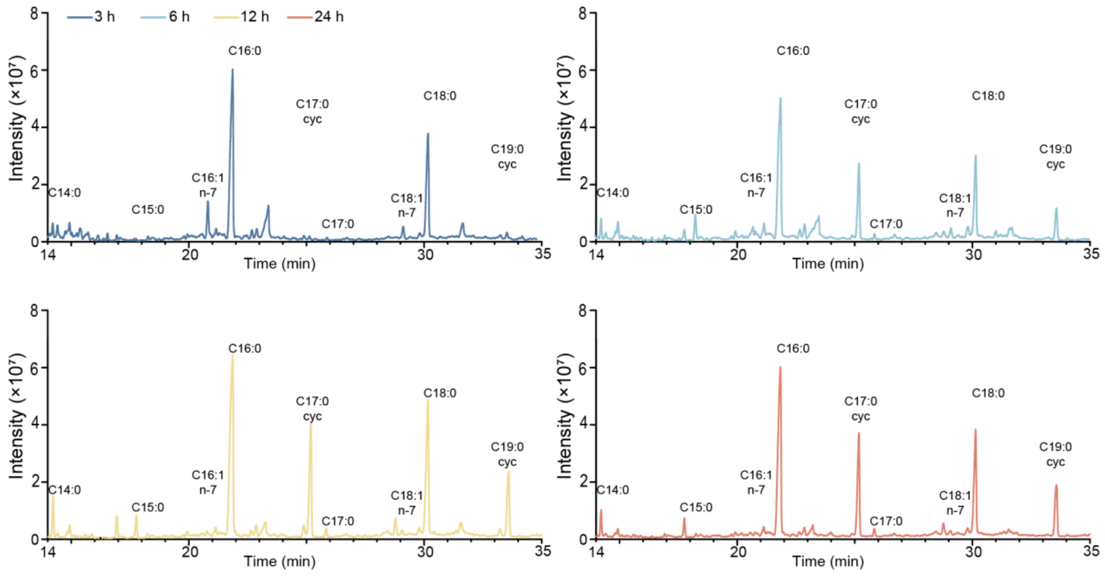


**Supplementary Figure S3. GC-MS TIC chromatograms of FAMEs from total lipid extracts of *E. coli* collected at 3, 6, 12, and 24 h.** Major fatty acids are annotated (C14:0, C15:0, C16:1 n-7, C16:0, C17:0, C17:0 cyc, C18:1 n-7, C18:0, and C19:0 cyc).

**Supplementary Table S1. RT-qPCR primers used in this study.**

| Genes | Sequence (5'→3') | Product (bp) |
|---|---|---|
| *16S rRNA* | CATGCCGCGTGTATGAAGAA<br>CGGGTAACGTCAATGAGCAAA | 96 |
| *pgsA* | GCTACTGCGTTTACCCAACTCC<br>GTTTGGTGCTTTCCTTGACCC | 178 |
| *pgpA* | AAACGGCGAAAGACATGGGT<br>CCACGGCTTCCACATATCCA | 158 |
| *psd* | TCAATGCTGCCAACAATCGTC<br>AATCTGTTTGCCCGTAACGAA | 101 |
| *pssA* | AGCCTGAACGATGTTTACCTG<br>TCGTTCTTGATTTCCGGGCTT | 182 |
| *clsA* | GTGTGAATGGTGTGACCGCT<br>GACTGGGAGATTGAAACCGGAA | 95 |
| *clsB* | CGCTTTTGGTCAGGTTCCAC<br>CCAGTAATCTCGATCCGCTCA | 177 |
| *clsC* | GTGACCCTGGTGGGAGGA<br>GCACCTGCTGTAAGGGTGAA | 169 |
| *fabA* | ACGACCATCAACCAGCACTT<br>TAGGGTTCTACCTCGGCTGG | 188 |
| *fabB* | CCATCTTCATGCAGCGTACT<br>CGTACTTACGACGCTCACCG | 193 |
| *cfa* | CTACGCCCGCATCTTGTTTG<br>CGAGCTAATGCGTAATGCCG | 185 |

**Supplementary Table S2. Phospholipid ratios (%) of *E. coli* at 12 h.**

| Phospholipid | ratio | Phospholipid | ratio | Phospholipid | ratio |
|---|---|---|---|---|---|
| PE(28:0-14:0/14:0) | 0.25 | PG(36:1-18:0/18:1) | 0.13 | CL(69:3-33:1/36:2) | 0.01 |
| PE(30:0-14:0/16:0) | 4.79 | PG(36:2-18:1/18:1) | 0.16 | CL(71:4-36:2/35:3) | 0.09 |
| PE(30:1-14:0/16:1) | 0.05 | PG(36:3-18:1/18:2) | 0.11 | CL(67:3-33:1/34:2) | 0.00 |
| PE(30:1-14:1/16:0) | 0.18 | PG(38:0 cyc-19:0 cyc/19:0 cyc) | 2.00 | CL(68:1-33:0/35:1) | 0.04 |
| PE(31:0-15:0/16:0) | 0.15 | CL(67:1-33:0/34:1) | 0.03 | CL(68:1-33:1/35:0) | 0.05 |
| PE(31:0 cyc-14:0/17:0 cyc) | 0.86 | CL(67:2-33:1/34:1) | 0.56 | CL(68:2-33:1/35:1) | 0.06 |
| PE(32:0-14:0/18:0) | 0.09 | CL(71:2-35:1/36:1) | 0.11 | CL(69:2-33:1/36:1) | 0.56 |
| PE(32:0-16:0/16:0) | 2.36 | CL(64:3-32:2/32:1) | 0.02 | CL(70:2-33:0/37:2) | 0.07 |
| PE(32:1-14:0/18:1) | 0.15 | CL(66:4-34:2/32:2) | 0.01 | CL(70:2-33:1/37:1) | 0.00 |
| PE(32:1-16:0/16:1) | 0.90 | CL(64:2-34:1/30:1) | 0.01 | CL(70:3-33:1/37:2) | 0.02 |
| PE(32:2-16:1/16:1) | 0.05 | CL(64:1-32:0/32:1) | 0.05 | CL(70:3-35:1/35:2) | 0.05 |
| PE(33:0-16:0/17:0) | 0.53 | CL(64:2-32:1/32:1) | 0.38 | CL(67:1-32:0/35:1) | 0.03 |
| PE(33:0 cyc-16:0/17:0 cyc) | 30.24 | CL(66:3-32:1/34:2) | 0.13 | CL(69:2-35:1/34:1) | 0.03 |
| PE(33:1 cyc-16:1/17:0 cyc) | 0.29 | CL(66:3-32:2/34:1) | 0.01 | CL(70:1-34:0/36:1) | 0.07 |
| PE(34:0-16:0/18:0) | 0.14 | CL(66:2-32:0/34:2) | 0.01 | CL(72:4-36:2/36:2) | 0.00 |
| PE(34:1-16:0/18:1) | 1.67 | CL(68:4-34:2/34:1) | 0.04 | CL(71:3-35:1/36:2) | 0.03 |
| PE(34:1-16:1/18:0) | 0.11 | CL(64:2-33:1/34:2) | 0.01 | CL(71:3-33:1/38:2) | 0.10 |
| PE(34:2-16:0/18:2) | 0.20 | CL(63:1-33:1/30:0) | 0.12 | CL(72:2-36:1/36:1) | 0.08 |
| PE(34:2-16:1/18:1) | 0.10 | CL(65:2-32:1/33:1) | 0.05 | CL(69:3-34:2/35:1) | 0.00 |
| PE(34:0 cyc-17:0 cyc/17:0 cyc) | 2.34 | CL(64:1-30:0/34:1) | 0.06 | CL(70:1-35:0/35:1) | 0.04 |
| PE(35:0 cyc-16:0/19:0 cyc) | 16.30 | CL(67:3-32:1/35:2) | 0.01 | CL(70:2-35:1/35:1) | 0.07 |
| PE(35:1 cyc-17:0 cyc/18:1) | 0.52 | CL(66:1-32:0/34:1) | 0.03 | CL(72:3-37:2/35:1) | 0.42 |
| PE(36:1-18:0/18:1) | 0.12 | CL(66:2-32:1/34:1) | 0.34 | CL(74:4-38:2/36:2) | 0.04 |
| PE(36:0 cyc-17:0/19:0 cyc) | 0.22 | CL(68:2-32:0/36:2) | 0.01 | CL(73:3-38:2/35:1) | 0.00 |
| PE(36:2-18:1/18:1) | 0.15 | CL(68:2-34:0/34:2) | 0.01 | | |
| PE(36:0 cyc-17:0 cyc/19:0 cyc) | 9.38 | CL(68:3-34:2/34:1) | 0.11 | | |
| PE(37:0 cyc-18:0/19:0 cyc) | 0.39 | CL(68:3-32:1/36:2) | 0.08 | | |
| PE(37:1 cyc-18:1/19:0 cyc) | 0.51 | CL(70:4-34:2/36:2) | 0.03 | | |
| PE(38:0 cyc-19:0/19:0 cyc) | 0.27 | CL(66:1-33:0/33:1) | 0.10 | | |
| PG(28:0-14:0/14:0) | 0.18 | CL(66:2-33:1/33:1) | 0.84 | | |

| | | | |
|---|---|---|---|
| PG(30:0-14:0/16:0) | 1.29 | CL(65:1-30:0/35:1) | 0.07 |
| PG(32:0-16:0/16:0) | 0.60 | CL(68:3-33:1-35:2) | 0.05 |
| PG(32:1-14:0/18:1) | 0.11 | CL(70:4-35:2/35:2) | 0.00 |
| PG(32:1-16:0/16:1) | 0.32 | CL(66:1-31:0/35:1) | 0.00 |
| PG(33:0-16:0/17:0) | 0.37 | CL(67:1-33:1/35:0) | 0.00 |
| PG(33:0 cyc-16:0/17:0 cyc) | 8.73 | CL(67:2-32:1/35:1) | 0.03 |
| PG(34:0-16:0/18:0) | 0.48 | CL(69:3-34:1/35:2) | 0.01 |
| PG(34:1-16:0/18:1) | 0.48 | CL(68:1-32:0/34:1) | 0.04 |
| PG(34:1-16:1/18:0) | 0.10 | CL(68:1-32:1/34:0) | 0.02 |
| PG(34:2-16:1/18:1) | 0.10 | CL(68:2-34:1/34:1) | 0.22 |
| PG(35:0 cyc-16:0/19:0 cyc) | 5.76 | CL(70:3-34:1/36:2) | 0.05 |
| PG(35:1 cyc-17:0 cyc/18:1) | 0.20 | CL(68:2-32:1/36:1) | 0.02 |

**Supplementary Table S3. Phospholipid ratios (%) of *E. coli* at different growth stages.**

| Phospholipids | 3h | 6h | 12h | 24h |
|---|---|---|---|---|
| PE(28:0-14:0/14:0) | 0.15 | 0.38 | 0.55 | 0.53 |
| PE(30:1-14:0/16:1) | 1.64 | 0.31 | 0.11 | 0.06 |
| PE(30:1-14:1/16:0) | 0.41 | 0.00 | 0.31 | 0.31 |
| PE(31:0 cyc-14:0/17:0 cyc) | 0.05 | 0.83 | 1.33 | 1.37 |
| PE(33:1 cyc-16:1/17:0 cyc) | 0.34 | 0.38 | 0.35 | 0.36 |
| PE(30:0-14:0/16:0) | 4.46 | 5.55 | 6.44 | 6.50 |
| PE(32:1-14:0/18:1) | 0.31 | 0.41 | 0.40 | 0.40 |
| PE(32:1-16:0/16:1) | 20.58 | 4.41 | 1.80 | 1.08 |
| PE(34:2-16:1/18:1) | 7.54 | 1.06 | 0.30 | 0.19 |
| PE(31:0-15:0/16:0) | 0.07 | 0.01 | 0.02 | 0.03 |
| PE(34:0 cyc-17:0 cyc/17:0 cyc) | 0.07 | 2.43 | 2.90 | 2.89 |
| PE(33:0 cyc-16:0/17:0 cyc) | 5.29 | 24.88 | 30.08 | 30.91 |
| PE(33:0-16:0/17:0) | 0.09 | 0.48 | 0.59 | 0.59 |
| PE(35:1 cyc-17:0 cyc/18:1) | 0.60 | 4.94 | 4.60 | 3.80 |
| PE(32:0-16:0/16:0) | 2.31 | 1.27 | 1.60 | 1.68 |
| PE(32:0-14:0/18:0) | 0.02 | 0.01 | 0.01 | 0.02 |
| PE(34:1-16:0/18:1) | 14.01 | 9.60 | 6.44 | 4.79 |
| PE(34:1-16:1/18:0) | 0.95 | 0.23 | 0.08 | 0.06 |
| PE(36:2-18:1/18:1) | 5.06 | 3.43 | 1.87 | 1.27 |
| PE(36:0 cyc-17:0/19:0 cyc) | 0.00 | 0.02 | 0.07 | 0.11 |
| PE(36:0 cyc-17:0 cyc/19:0 cyc) | 0.02 | 2.45 | 5.65 | 6.85 |
| PE(37:1 cyc-18:1/19:0 cyc) | 0.05 | 1.98 | 2.74 | 2.66 |
| PE(34:0-16:0/18:0) | 0.31 | 0.21 | 0.12 | 0.09 |
| PE(36:1-18:0/18:1) | 0.45 | 0.31 | 0.19 | 0.16 |
| PE(37:0 cyc-18:0/19:0 cyc) | 0.00 | 0.16 | 0.29 | 0.35 |
| PG(28:0-14:0/14:0) | 0.03 | 0.10 | 0.11 | 0.09 |
| PG(30:0-14:0/16:0) | 1.07 | 1.21 | 1.20 | 1.13 |
| PG(32:1-14:0/18:1) | 0.19 | 0.20 | 0.14 | 0.11 |
| PG(32:1-16:0/16:1) | 8.61 | 1.49 | 0.62 | 0.62 |
| PG(34:2-16:1/18:1) | 3.70 | 0.87 | 0.29 | 0.25 |
| PG(33:0-16:0/17:0) | 0.02 | 0.16 | 0.17 | 0.17 |
| PG(33:0 cyc-16:0/17:0 cyc) | 1.27 | 9.34 | 9.78 | 10.14 |
| PG(35:1 cyc-17:0 cyc/18:1) | 0.12 | 1.32 | 0.97 | 0.86 |
| PG(32:0-16:0/16:0) | 0.71 | 0.44 | 0.41 | 0.42 |
| PG(34:0-16:0/18:0) | 0.19 | 0.11 | 0.06 | 0.05 |
| PG(34:1-16:0/18:1) | 7.61 | 4.56 | 2.45 | 2.43 |
| PG(36:2-18:1/18:1) | 4.82 | 2.93 | 1.43 | 1.22 |
| PG(36:0 cyc-17:0/19:0 cyc) | 0.00 | 0.02 | 0.03 | 0.03 |
| PG(35:0 cyc-16:0/19:0 cyc) | 0.36 | 5.39 | 6.01 | 6.05 |
| PG(36:1-18:0/18:1) | 0.43 | 0.31 | 0.16 | 0.15 |
| PG(30:1-14:1/16:0) | 0.42 | 0.24 | 0.29 | 0.32 |
| PG(38:0 cyc-19:0 cyc/19:0 cyc) | 0.00 | 0.57 | 1.36 | 1.43 |

| | | | | |
|---|---|---|---|---|
| PG(33:1 cyc-16:1/17:0 cyc) | 0.07 | 0.09 | 0.07 | 0.06 |
| CL(71:2-35:1/36:1) | 0.00 | 0.04 | 0.07 | 0.10 |
| CL(60:2-30:1/30:1) | 0.00 | 0.00 | 0.00 | 0.00 |
| CL(64:3-32:2/32:1) | 0.12 | 0.01 | 0.00 | 0.01 |
| CL(64:2-34:1/30:1) | 0.03 | 0.01 | 0.01 | 0.01 |
| CL(64:1-32:0/32:1) | 0.29 | 0.09 | 0.06 | 0.08 |
| CL(64:2-32:1/32:1) | 1.56 | 0.37 | 0.24 | 0.34 |
| CL(66:2-32:0/34:2) | 0.09 | 0.02 | 0.01 | 0.02 |
| CL(66:3-32:1/34:2) | 0.54 | 0.10 | 0.05 | 0.07 |
| CL(68:4-34:2/34:2) | 0.22 | 0.04 | 0.02 | 0.03 |
| CL(64:2-33:1/34:2) | 0.00 | 0.02 | 0.04 | 0.07 |
| CL(63:1-33:1/30:0) | 0.01 | 0.11 | 0.18 | 0.24 |
| CL(65:2-32:1/33:1) | 0.08 | 0.17 | 0.11 | 0.14 |
| CL(64:1-30:0/34:1) | 0.08 | 0.07 | 0.05 | 0.06 |
| CL(66:4-34:2/32:2) | 0.03 | 0.00 | 0.00 | 0.00 |
| CL(67:3-32:1/35:2) | 0.02 | 0.04 | 0.02 | 0.02 |
| CL(66:2-32:1/34:1) | 0.80 | 0.28 | 0.17 | 0.20 |
| CL(68:2-34:0/34:2) | 0.03 | 0.01 | 0.00 | 0.01 |
| CL(68:3-34:2/34:1) | 0.24 | 0.09 | 0.05 | 0.05 |
| CL(68:3-32:1/36:2) | 0.25 | 0.08 | 0.04 | 0.05 |
| CL(68:2-32:1/36:1) | 0.07 | 0.04 | 0.03 | 0.03 |
| CL(70:4-34:2/36:2) | 0.09 | 0.03 | 0.02 | 0.03 |
| CL(67:3-33:1/34:2) | 0.03 | 0.12 | 0.08 | 0.13 |
| CL(66:2-33:1/33:1) | 0.01 | 0.59 | 0.93 | 1.32 |
| CL(66:1-33:0/33:1) | 0.00 | 0.08 | 0.12 | 0.17 |
| CL(68:3-33:1-35:2) | 0.00 | 0.13 | 0.16 | 0.18 |
| CL(65:1-30:0/35:1) | 0.00 | 0.05 | 0.10 | 0.13 |
| CL(66:1-31:0/35:1) | 0.00 | 0.00 | 0.00 | 0.01 |
| CL(70:4-35:2/35:2) | 0.00 | 0.01 | 0.01 | 0.01 |
| CL(66:3-32:2/34:1) | 0.05 | 0.01 | 0.00 | 0.00 |
| CL(67:1-33:1/35:0) | 0.00 | 0.01 | 0.01 | 0.01 |
| CL(67:1-33:0/34:1) | 0.01 | 0.03 | 0.02 | 0.02 |
| CL(67:2-33:1/34:1) | 0.06 | 0.32 | 0.27 | 0.32 |
| CL(67:2-32:1/35:1) | 0.02 | 0.08 | 0.06 | 0.08 |
| CL(69:3-34:1/35:2) | 0.01 | 0.05 | 0.04 | 0.04 |
| CL(66:1-32:0/34:1) | 0.11 | 0.05 | 0.03 | 0.04 |
| CL(68:2-32:0/36:2) | 0.04 | 0.02 | 0.01 | 0.02 |
| CL(70:1-34:0/36:1) | 0.00 | 0.00 | 0.00 | 0.00 |
| CL(68:2-34:1/34:1) | 0.41 | 0.24 | 0.12 | 0.14 |
| CL(70:3-34:1/36:2) | 0.21 | 0.12 | 0.06 | 0.07 |
| CL(68:4-32:2/36:2) | 0.01 | 0.00 | 0.00 | 0.00 |
| CL(69:3-33:1/36:2) | 0.02 | 0.15 | 0.20 | 0.28 |
| CL(69:3-34:2/35:1) | 0.00 | 0.04 | 0.05 | 0.06 |
| CL(71:4-36:2/35:3) | 0.00 | 0.03 | 0.03 | 0.03 |

| | | | | |
|---|---|---|---|---|
| CL(68:1-33:0/35:1) | 0.00 | 0.04 | 0.07 | 0.09 |
| CL(68:2-33:1/35:1) | 0.01 | 0.41 | 0.75 | 0.97 |
| CL(70:2-33:1/37:1) | 0.00 | 0.03 | 0.05 | 0.07 |
| CL(70:3-33:1/37:2) | 0.00 | 0.06 | 0.09 | 0.11 |
| CL(67:1-32:0/35:1) | 0.00 | 0.03 | 0.04 | 0.05 |
| CL(70:3-35:1/35:2) | 0.00 | 0.06 | 0.09 | 0.10 |
| CL(69:2-33:1/36:1) | 0.00 | 0.09 | 0.14 | 0.18 |
| CL(70:2-33:0/37:2) | 0.00 | 0.00 | 0.01 | 0.01 |
| CL(69:2-35:1/34:1) | 0.00 | 0.12 | 0.11 | 0.13 |
| CL(72:4-36:2/36:2) | 0.05 | 0.04 | 0.04 | 0.05 |
| CL(71:3-35:1/36:2) | 0.00 | 0.08 | 0.14 | 0.19 |
| CL(71:3-33:1/38:2) | 0.00 | 0.01 | 0.04 | 0.06 |
| CL(74:3-38:1/36:2) | 0.00 | 0.00 | 0.00 | 0.00 |
| CL(70:1-35:0/35:1) | 0.00 | 0.03 | 0.07 | 0.10 |
| CL(70:2-35:1/35:1) | 0.00 | 0.15 | 0.37 | 0.54 |
| CL(72:3-37:2/35:1) | 0.00 | 0.03 | 0.06 | 0.08 |
| CL(72:2-36:1/36:1) | 0.00 | 0.00 | 0.00 | 0.00 |
| CL(74:4-38:2/36:2) | 0.00 | 0.00 | 0.01 | 0.02 |
| CL(73:3-38:2/35:1) | 0.00 | 0.00 | 0.02 | 0.05 |

**Supplementary Table S4. Comparison of phospholipid class proportions determined by TLC densitometry and LC-MS/MS across growth stages in *E. coli*.**

| Time (h) | 3 | | 6 | | 12 | | 24 | |
|---|---|---|---|---|---|---|---|---|
| | TLC | LC-MS/MS | TLC | LC-MS/MS | TLC | LC-MS/MS | TLC | LC-MS/MS |
| CL | 4.52 | 5.60 | 5.25 | 4.93 | 7.66 | 5.57 | 10.26 | 7.42 |
| PG | 18.63 | 29.55 | 23.31 | 29.26 | 24.05 | 25.48 | 17.08 | 25.47 |
| PE | 76.85 | 64.78 | 71.44 | 65.74 | 68.29 | 68.84 | 72.66 | 67.06 |

**Supplementary Table S5. Phospholipid ratios (%) of *E. coli* under sub-inhibitory antibiotics.**

| Phospholipids | PBS | Ampicillin | Triclosan | Kanamycin | Ciprofloxacin | Colistin |
|---|---|---|---|---|---|---|
| PG(28:0-14:0/14:0) | 0.00 | 0.04 | 0.16 | 0.24 | 0.30 | 0.00 |
| PG(30:1-14:0/16:1) | 0.00 | 0.00 | 0.00 | 0.00 | 0.03 | 0.00 |
| PG(31:0 cyc -14:0/17:0 cyc) | 0.20 | 0.44 | 0.81 | 0.81 | 0.78 | 0.29 |
| PG(30:0-14:0/16:0) | 0.50 | 1.02 | 1.79 | 2.23 | 2.51 | 0.34 |
| PG(32:1-14:0/18:1) | 0.01 | 0.01 | 0.00 | 0.00 | 0.01 | 0.00 |
| PG(32:1-16:0/16:1) | 0.29 | 0.21 | 0.16 | 0.15 | 0.33 | 0.11 |
| PG(34:2-16:1/18:1) | 0.04 | 0.04 | 0.00 | 0.00 | 0.04 | 0.01 |
| PG(34:0 cyc-17:0 cyc/17:0 cyc) | 0.44 | 0.38 | 0.45 | 0.43 | 0.31 | 0.00 |
| PG(33:0 cyc -14:0/19:0 cyc) | 0.08 | 0.08 | 0.08 | 0.12 | 0.10 | 0.15 |
| PG(33:0 cyc -16:0/17:0 cyc) | 7.34 | 9.45 | 9.59 | 9.50 | 9.44 | 10.14 |
| PG(32:0-16:0/16:0) | 0.42 | 0.78 | 0.90 | 1.19 | 1.19 | 1.67 |
| PG(35:1 cyc-17:0 cyc/18:1) | 0.31 | 0.16 | 0.20 | 0.19 | 0.08 | 0.12 |

| | | | | | | |
|---|---|---|---|---|---|---|
| PG(35:1 cyc - 16:1/19:0 cyc) | 0.06 | 0.02 | 0.00 | 0.00 | 0.00 | 0.02 |
| PG(35:0 cyc - 16:0/19:0 cyc) | 6.22 | 3.83 | 3.46 | 4.18 | 4.80 | 9.59 |
| PG(34:1- 16:0/18:1) | 1.01 | 0.00 | 0.00 | 0.00 | 0.45 | 0.00 |
| PG(34:1- 16:1/18:0) | 0.00 | 0.20 | 0.27 | 0.31 | 0.00 | 0.40 |
| PG(36:2- 18:1/18:1) | 0.22 | 0.15 | 0.02 | 0.06 | 0.09 | 0.16 |
| PG(36:0 cyc- 17:0 cyc/19:0 cyc) | 1.28 | 1.00 | 0.97 | 1.03 | 0.92 | 1.73 |
| PG(37:1 cyc - 18:1/19:0 cyc) | 0.37 | 0.20 | 0.14 | 0.15 | 0.08 | 0.17 |
| PG(34:0- 16:0/18:0) | 0.05 | 0.03 | 0.00 | 0.00 | 0.02 | 0.04 |
| PG(36:1- 18:0/18:1) | 0.09 | 0.02 | 0.00 | 0.00 | 0.00 | 0.04 |
| PG(38:0 cyc- 19:0 cyc/19:0 cyc) | 0.98 | 0.61 | 0.48 | 0.66 | 0.71 | 1.35 |
| PG(37:0 cyc - 18:0/19:0 cyc) | 0.08 | 0.05 | 0.02 | 0.02 | 0.03 | 0.16 |
| PG(33:1 cyc - 16:1/17:0 cyc) | 0.00 | 0.01 | 0.03 | 0.01 | 0.00 | 0.12 |
| PE(28:0- 14:0/14:0) | 0.29 | 0.59 | 0.99 | 1.21 | 1.19 | 0.12 |

| | | | | | | |
|---|---|---|---|---|---|---|
| PE(30:1-14:0/16:1) | 0.04 | 0.02 | 0.00 | 0.00 | 0.00 | 0.00 |
| PE(32:2-16:1/16:1) | 0.00 | 0.02 | 0.00 | 0.00 | 0.00 | 0.00 |
| PE(31:0 cyc -14:0/17:0 cyc) | 1.62 | 2.02 | 2.58 | 2.47 | 1.08 | 1.04 |
| PE(30:0-14:0/16:0) | 4.83 | 5.98 | 7.72 | 8.27 | 9.16 | 2.32 |
| PE(33:1 cyc -16:1/17:0 cyc) | 0.31 | 0.13 | 0.38 | 0.12 | 0.11 | 0.09 |
| PE(32:1-14:0/18:1) | 0.22 | 0.03 | 0.00 | 0.00 | 0.01 | 0.03 |
| PE(34:2-16:1/18:1) | 0.17 | 0.11 | 0.00 | 0.00 | 0.04 | 0.05 |
| PE(32:1-16:0/16:1) | 1.97 | 1.01 | 0.31 | 0.16 | 0.78 | 0.38 |
| PE(34:0 cyc-17:0 cyc/17:0 cyc) | 2.48 | 2.38 | 2.80 | 2.38 | 1.59 | 1.18 |
| PE(33:0 cyc -14:0/19:0 cyc) | 0.33 | 0.39 | 0.53 | 0.52 | 0.39 | 0.33 |
| PE(33:0 cyc -16:0/17:0 cyc) | 32.05 | 37.63 | 35.56 | 33.34 | 29.50 | 33.21 |
| PE(32:0-16:0/16:0) | 2.21 | 3.23 | 2.85 | 2.94 | 2.96 | 4.43 |
| PE(35:1 cyc -17:0 cyc/18:1) | 2.68 | 0.47 | 0.48 | 0.39 | 0.32 | 0.49 |
| PE(35:1 cyc -16:1/19:0 cyc) | 0.08 | 0.01 | 0.02 | 0.00 | 0.00 | 0.00 |

| | | | | | | |
|---|---|---|---|---|---|---|
| PE(34:1-16:0/18:1) | 2.81 | 0.95 | 0.56 | 0.45 | 0.40 | 1.60 |
| PE(34:1-16:1/18:0) | 0.03 | 0.00 | 0.00 | 0.00 | 0.00 | 0.01 |
| PE(36:2-18:1/18:1) | 0.54 | 0.12 | 0.00 | 0.00 | 0.03 | 0.20 |
| PE(36:0 cyc-17:0 cyc/19:0 cyc) | 4.99 | 3.99 | 5.05 | 5.23 | 3.89 | 3.63 |
| PE(35:0 cyc -16:0/19:0 cyc) | 8.12 | 6.37 | 6.14 | 6.46 | 6.28 | 10.15 |
| PE(35:0 cyc -18:0/17:0 cyc) | 0.55 | 0.55 | 0.46 | 0.46 | 0.37 | 0.75 |
| PE(34:0-16:0/18:0) | 0.10 | 0.06 | 0.02 | 0.02 | 0.03 | 0.23 |
| PE(37:1 cyc -18:1/19:0 cyc) | 1.80 | 0.30 | 0.25 | 0.23 | 0.11 | 0.72 |
| PE(36:1-18:0/18:1) | 0.03 | 0.00 | 0.00 | 0.00 | 0.00 | 0.02 |
| PE(38:0 cyc-19:0 cyc/19:0 cyc) | 2.97 | 2.37 | 2.48 | 3.21 | 2.75 | 2.94 |
| PE(37:0 cyc -18:0/19:0 cyc) | 0.23 | 0.12 | 0.07 | 0.08 | 0.08 | 0.32 |
| CL(64:2-34:1/30:1) | 0.01 | 0.01 | 0.01 | 0.01 | 0.01 | 0.00 |
| CL(64:2-32:1/32:1) | 0.24 | 0.66 | 0.19 | 0.14 | 0.62 | 0.11 |

| | | | | | | |
|---|---|---|---|---|---|---|
| CL(66:3-32:1/34:2) | 0.05 | 0.11 | 0.05 | 0.04 | 0.12 | 0.01 |
| CL(68:4-34:2/34:2) | 0.03 | 0.02 | 0.02 | 0.01 | 0.03 | 0.00 |
| CL(64:2-33:1/34:2) | 0.07 | 0.28 | 0.34 | 0.32 | 0.41 | 0.03 |
| CL(63:1-33:1/30:0) | 0.22 | 0.86 | 0.92 | 0.99 | 1.58 | 0.06 |
| CL(64:1-30:0/34:1) | 0.06 | 0.12 | 0.08 | 0.08 | 0.15 | 0.01 |
| CL(64:1-32:0/32:1) | 0.07 | 0.16 | 0.08 | 0.09 | 0.20 | 0.03 |
| CL(66:2-32:1/34:1) | 0.18 | 0.29 | 0.09 | 0.09 | 0.39 | 0.12 |
| CL(67:3-32:1/35:2) | 0.02 | 0.02 | 0.02 | 0.01 | 0.01 | 0.00 |
| CL(64:3-32:2/32:1) | 0.01 | 0.02 | 0.05 | 0.04 | 0.03 | 0.00 |
| CL(65:2-32:1/33:1) | 0.18 | 0.52 | 0.74 | 0.62 | 0.63 | 0.00 |
| CL(68:3-34:2/34:1) | 0.02 | 0.05 | 0.01 | 0.01 | 0.05 | 0.01 |
| CL(67:3-33:1/34:2) | 0.08 | 0.14 | 0.19 | 0.13 | 0.12 | 0.12 |
| CL(66:2-33:1/33:1) | 1.80 | 3.58 | 3.14 | 2.88 | 4.53 | 2.30 |
| CL(66:1-33:0/33:1) | 0.22 | 0.46 | 0.37 | 0.35 | 0.55 | 0.30 |
| CL(66:2-32:0/34:2) | 0.01 | 0.02 | 0.01 | 0.01 | 0.02 | 0.01 |
| CL(68:3-33:1/35:2) | 0.16 | 0.07 | 0.10 | 0.06 | 0.07 | 0.06 |

| | | | | | | |
|---|---|---|---|---|---|---|
| CL(65:1-30:0/35:1) | 0.13 | 0.15 | 0.18 | 0.24 | 0.48 | 0.05 |
| CL(66:1-32:0/34:1) | 0.03 | 0.07 | 0.03 | 0.03 | 0.07 | 0.05 |
| CL(68:1-32:0/34:1) | 0.02 | 0.04 | 0.01 | 0.01 | 0.03 | 0.03 |
| CL(68:2-34:1/34:1) | 0.13 | 0.14 | 0.05 | 0.05 | 0.16 | 0.14 |
| CL(69:2-33:1/36:1) | 0.24 | 0.34 | 0.28 | 0.28 | 0.37 | 0.17 |
| CL(70:2-33:1/37:1) | 0.05 | 0.12 | 0.05 | 0.06 | 0.09 | 0.08 |
| CL(68:1-32:1/34:0) | 0.01 | 0.02 | 0.00 | 0.00 | 0.02 | 0.01 |
| CL(68:3-32:1/36:2) | 0.03 | 0.08 | 0.10 | 0.09 | 0.06 | 0.01 |
| CL(69:3-34:1/35:2) | 0.04 | 0.01 | 0.01 | 0.01 | 0.01 | 0.01 |
| CL(67:2-32:1/35:1) | 0.13 | 0.16 | 0.22 | 0.22 | 0.27 | 0.07 |
| CL(67:1-33:1/34:0) | 0.01 | 0.02 | 0.03 | 0.03 | 0.03 | 0.01 |
| CL(68:2-32:1/36:1) | 0.01 | 0.09 | 0.03 | 0.03 | 0.02 | 0.01 |
| CL(66:3-32:2/34:1) | 0.00 | 0.01 | 0.00 | 0.00 | 0.01 | 0.00 |
| CL(67:2-33:1/34:1) | 0.50 | 0.34 | 0.58 | 0.50 | 0.74 | 0.29 |
| CL(66:1-31:0/35:1) | 0.03 | 0.03 | 0.04 | 0.05 | 0.07 | 0.00 |
| CL(70:4-34:2/36:2) | 0.01 | 0.02 | 0.02 | 0.02 | 0.01 | 0.01 |

| | | | | | | |
|---|---|---|---|---|---|---|
| CL(69:3-33:1/36:2) | 0.28 | 0.42 | 0.50 | 0.49 | 0.56 | 0.26 |
| CL(69:3-34:2/35:1) | 0.04 | 0.04 | 0.05 | 0.04 | 0.04 | 0.09 |
| CL(68:2-32:0/36:2) | 0.01 | 0.03 | 0.02 | 0.03 | 0.04 | 0.02 |
| CL(68:2-33:1/35:1) | 1.20 | 1.28 | 1.11 | 1.12 | 1.91 | 1.71 |
| CL(68:1-33:0/35:1) | 0.12 | 0.15 | 0.13 | 0.13 | 0.20 | 0.20 |
| CL(70:3-33:1/37:2) | 0.13 | 0.05 | 0.07 | 0.05 | 0.06 | 0.07 |
| CL(67:1-32:0/35:1) | 0.05 | 0.06 | 0.05 | 0.06 | 0.13 | 0.17 |
| CL(70:3-35:1/35:2) | 0.12 | 0.02 | 0.04 | 0.02 | 0.03 | 0.05 |
| CL(70:3-34:1/36:2) | 0.08 | 0.07 | 0.06 | 0.05 | 0.06 | 0.02 |
| CL(69:2-35:1/34:1) | 0.15 | 0.16 | 0.08 | 0.08 | 0.09 | 0.15 |
| CL(72:4-36:2/36:2) | 0.06 | 0.05 | 0.08 | 0.08 | 0.05 | 0.02 |
| CL(71:3-35:1/36:2) | 0.22 | 0.15 | 0.19 | 0.20 | 0.21 | 0.17 |
| CL(71:3-33:1/38:2) | 0.16 | 0.21 | 0.24 | 0.28 | 0.33 | 0.16 |
| CL(71:2-35:1/36:1) | 0.10 | 0.09 | 0.05 | 0.03 | 0.09 | 0.09 |
| CL(70:1-34:0/36:1) | 0.00 | 0.00 | 0.00 | 0.00 | 0.00 | 0.19 |
| CL(70:2-35:1/35:1) | 0.77 | 0.48 | 0.38 | 0.44 | 0.74 | 1.29 |

| | | | | | | |
|---|---|---|---|---|---|---|
| CL(72:3-37:2/35:1) | 0.10 | 0.02 | 0.02 | 0.02 | 0.02 | 0.05 |
| CL(72:2-36:1/36:1) | 0.02 | 0.01 | 0.01 | 0.01 | 0.01 | 0.00 |
| CL(74:4-38:2/36:2) | 0.04 | 0.03 | 0.04 | 0.05 | 0.04 | 0.01 |
| CL(74:3-38:1/36:2) | 0.01 | 0.00 | 0.01 | 0.01 | 0.00 | 0.00 |
| CL(73:3-38:2/35:1) | 0.11 | 0.08 | 0.07 | 0.11 | 0.13 | 0.10 |
| CL(67:1-33:0/34:1) | 0.00 | 0.00 | 0.00 | 0.00 | 0.00 | 0.01 |
| CL(68:4-32:2/36:2) | 0.00 | 0.00 | 0.00 | 0.00 | 0.00 | 0.01 |
| CL(68:1-33:1/35:0) | 0.00 | 0.00 | 0.00 | 0.00 | 0.00 | 0.21 |

**Supplementary Table S6. Phospholipid ratios (%) of VSE and VRE.**

| Phospholipids | VSE | VRE |
|---|---|---|
| PG(34:3-16:1/18:2) | 0.00 | 0.52 |
| PG(30:1-14:0/16:1) | 3.63 | 3.02 |
| PG(30:0-14:0/16:0) | 19.83 | 1.53 |
| PG(32:1-14:0/18:1) | 3.01 | 15.58 |
| PG(32:1-16:0/16:1) | 3.37 | 2.03 |
| PG(34:2-16:1/18:1) | 0.93 | 20.76 |
| PG(34:2-16:0/18:2) | 0.37 | 0.43 |
| PG(33:1-15:0/18:1) | 0.00 | 1.09 |
| PG(33:0-14:0/19:0) | 1.47 | 0.00 |
| PG(32:0-16:0/16:0) | 3.84 | 0.93 |
| PG(35:1-16:1/19:0) | 1.67 | 0.00 |
| PG(42:0-21:0/21:0) | 5.42 | 0.00 |
| PG(32:0-14:0/18:0) | 1.03 | 0.12 |
| PG(34:1-16:0/18:1) | 8.94 | 34.41 |
| PG(34:1-16:1/18:0) | 0.58 | 0.22 |
| PG(35:0-16:0/19:0) | 4.46 | 0.00 |
| PG(34:0-16:0/18:0) | 0.00 | 1.68 |
| PG(32:2-16:1/16:1) | 0.36 | 9.03 |
| PG(32:2-14:0/18:2) | 0.95 | 0.48 |
| PG(31:1-15:0/16:1) | 0.00 | 0.12 |
| PG(36:4-18:2/18:2) | 0.00 | 0.11 |
| PG(37:1-18:1/19:0) | 0.69 | 0.00 |
| PG(31:0-15:0/16:0) | 0.00 | 0.03 |
| PG(36:3-18:2/18:1) | 0.00 | 0.03 |
| PG(33:0-15:0/18:0) | 0.00 | 0.03 |
| PG(36:2-18:1/18:1) | 1.03 | 6.94 |
| PG(36:1-18:0/18:1) | 0.00 | 0.69 |
| CL(61:2-30:1/31:1) | 0.10 | 0.00 |
| CL(62:3-30:1/32:2) | 0.00 | 0.01 |
| CL(63:2-30:1/33:1) | 0.41 | 0.00 |
| CL(66:3-33:2/33:1) | 0.16 | 0.00 |
| CL(63:1-30:0/33:1) | 0.49 | 0.00 |
| CL(64:3-32:2/32:1) | 0.00 | 0.01 |
| CL(56:0-28:0/28:0) | 0.72 | 0.00 |
| CL(58:1-28:0/30:1) | 0.49 | 0.00 |
| CL(60:2-30:1/30:1) | 0.26 | 0.00 |
| CL(66:3-32:2/34:1) | 0.00 | 0.01 |
| CL(59:1-28:0/31:1) | 0.33 | 0.00 |
| CL(68:3-33:1/35:2) | 0.75 | 0.00 |
| CL(58:0-28:0/30:0) | 1.03 | 0.00 |
| CL(61:2-28:0/33:2) | 0.22 | 0.00 |
| CL(60:1-28:0/32:1) | 0.39 | 0.00 |

| | | |
|---|---|---|
| CL(60:1-30:0/30:1) | 0.44 | 0.00 |
| CL(62:2-28:0/34:2) | 0.31 | 0.00 |
| CL(62:2-30:1/32:1) | 0.18 | 0.02 |
| CL(67:3-32:2/35:1) | 0.24 | 0.00 |
| CL(61:1-28:0/33:1) | 2.10 | 0.00 |
| CL(61:1-30:0/31:1) | 0.10 | 0.00 |
| CL(61:0-28:0/33:0) | 0.14 | 0.00 |
| CL(60:0-30:0/30:0) | 0.17 | 0.00 |
| CL(60:0-28:0/32:0) | 0.08 | 0.00 |
| CL(62:1-28:0/34:1) | 0.82 | 0.00 |
| CL(63:2-28:0/35:2) | 0.74 | 0.00 |
| CL(62:1-30:0/32:1) | 0.27 | 0.00 |
| CL(64:2-32:1/32:1) | 0.09 | 0.03 |
| CL(65:3-30:1/35:2) | 0.16 | 0.00 |
| CL(64:2-30:1/34:1) | 0.13 | 0.00 |
| CL(64:2-31:1/33:1) | 0.22 | 0.00 |
| CL(63:1-28:0/35:1) | 2.09 | 0.00 |
| CL(65:2-32:1/33:1) | 0.36 | 0.00 |
| CL(65:2-30:0/35:2) | 0.28 | 0.00 |
| CL(65:2-31:1/34:1) | 0.13 | 0.00 |
| CL(68:4-33:2/35:2) | 0.21 | 0.00 |
| CL(67:3-32:1/35:2) | 0.30 | 0.00 |
| CL(67:3-33:2/34:1) | 0.25 | 0.00 |
| CL(66:2-32:1/34:1) | 0.65 | 0.07 |
| CL(67:3-30:1/37:2) | 0.07 | 0.00 |
| CL(67:3-34:2/33:1) | 0.13 | 0.00 |
| CL(66:2-33:1/33:1) | 1.48 | 0.00 |
| CL(68:1-33:1/35:0) | 0.68 | 0.00 |
| CL(66:2-31:1/35:1) | 0.71 | 0.00 |
| CL(65:1-30:0/35:1) | 1.38 | 0.00 |
| CL(68:3-33:2/35:1) | 0.30 | 0.00 |
| CL(67:2-32:1/35:1) | 0.87 | 0.00 |
| CL(67:2-33:1/34:1) | 0.52 | 0.00 |
| CL(70:4-35:2/35:2) | 0.60 | 0.00 |
| CL(69:3-34:1/35:2) | 0.50 | 0.00 |
| CL(66:1-32:0/34:1) | 0.20 | 0.00 |
| CL(68:2-34:1/34:1) | 0.51 | 0.04 |
| CL(69:3-32:1/37:2) | 0.12 | 0.00 |
| CL(68:1-33:0/35:1) | 0.21 | 0.00 |
| CL(68:2-33:1/35:1) | 2.45 | 0.00 |
| CL(67:1-32:0/35:1) | 0.27 | 0.00 |
| CL(70:3-35:1/35:2) | 1.51 | 0.00 |
| CL(70:3-33:1/37:2) | 0.31 | 0.00 |
| CL(69:2-34:1/35:1) | 2.96 | 0.00 |

| | | |
|---|---|---|
| CL(69:1-34:1/35:0) | 0.14 | 0.00 |
| CL(70:2-35:0/35:2) | 0.16 | 0.00 |
| CL(72:4-35:2/37:2) | 0.17 | 0.00 |
| CL(71:3-34:1/37:2) | 0.21 | 0.00 |
| CL(71:3-35:1/36:2) | 0.35 | 0.00 |
| CL(69:1-34:0/35:1) | 0.13 | 0.00 |
| CL(70:1-35:0/35:1) | 0.61 | 0.00 |
| CL(70:2-35:1/35:1) | 4.36 | 0.00 |
| CL(72:3-35:1/37:2) | 0.71 | 0.00 |
| CL(66:3-34:2/32:1) | 0.00 | 0.01 |
| CL(68:3-34:2/34:1) | 0.00 | 0.02 |
| CL(70:3-34:1/36:2) | 0.00 | 0.01 |
